# Development of novel cyclic NGR peptide–daunomycin conjugates with dual targeting property

**DOI:** 10.3762/bjoc.14.78

**Published:** 2018-04-25

**Authors:** Andrea Angelo Pierluigi Tripodi, Szilárd Tóth, Kata Nóra Enyedi, Gitta Schlosser, Gergely Szakács, Gábor Mező

**Affiliations:** 1 Eötvös Loránd University, Faculty of Science, Institute of Chemistry, Pázmány P. stny. 1/A, H-1117 Budapest, Hungary; 2 MTA-ELTE Research Group of Peptide Chemistry, Hungarian Academy of Sciences, Eötvös Loránd University, Pázmány P. stny. 1/A, H-1117 Budapest, Hungary; 3 Institute of Enzimology, Research Center for Natural Sciences, Hungarian Academy of Sciences, Magyar tudósok körútja 2, H-1117 Budapest, Hungary; 4 Institute of Cancer Research, Medical University Vienna, Borschkegasse 8a, A-1090 Vienna, Austria

**Keywords:** antitumor activity, drug release, NGR peptides, oxime-linkage, targeted drug delivery

## Abstract

Cyclic NGR peptides as homing devices are good candidates for the development of drug conjugates for targeted tumor therapy. In our previous study we reported that the Dau=Aoa-GFLGK(c[KNGRE]-GG-)-NH_2_ conjugate has a significant antitumor activity against both CD13+ HT-1080 human fibrosarcoma and CD13− but integrin positive HT-29 human colon adenocarcinoma cells. However, it seems that the free ε-amino group of Lys in the cycle is not necessary for the biological activity. Therefore, we developed novel cyclic NGR peptide–daunomycin conjugates in which Lys was replaced by different amino acids (Ala, Leu, Nle, Pro, Ser). The exchange of the Lys residue in the cycle simplified the cyclization step and resulted in a higher yield. The new conjugates showed lower chemostability against deamidation of Asn than the control compound, thus they had lower selectivity to CD13+ cells. However, the cellular uptake and cytotoxic effect of Dau=Aoa-GFLGK(c[NleNGRE]-GG-)-NH_2_ was higher in comparison to the control especially on HT-29 cells. Therefore, this conjugate is more suitable for drug targeting with dual targeting property.

## Introduction

Targeted chemotherapy is one of the most promising approaches for selective cancer treatment that may decrease the toxic side effects of anticancer drugs. This therapeutic approach is based on the fact that tumor specific receptors are highly expressed on cancer cells/tissues. NGR (Asn-Gly-Arg) motif-containing peptides identified by phage display are suitable candidates for selective drug delivery. NGR peptides bind to CD13-receptors on tumor cells and tumor related angiogenic blood vessels [1–2]. CD13 is a transmembrane zinc-dependent metalloprotease that functions in cell proliferation, cell migration and angiogenesis [1,3–4]. However, it is known that the Asn-Gly moiety is subject to Asn deamidation through succinimide formation leading to isoaspartic acid (isoAsp, isoD) and aspartic acid derivatives usually in a ratio of 3:1 after hydrolysis [5–11]. IsoDGR peptides are bound to RGD-integrin receptors with high affinity [12–14]. Due to their function in tumor proliferation, metastasis and angiogenesis, integrin receptors are also promising targets for cancer therapy. Thus, NGR-peptide homing devices may provide dual targeted delivery of anticancer drugs.

According to literature data, one of the most stable and tumor-selective cyclic NGR-peptides is c[KNGRE]-NH_2_, in which the α-amino group of the *N*-terminal Lys is coupled to the γ-carboxyl group of the glutamic acid residue (head-to-side chain cycle). In vitro fluorescence microscopy studies of an Oregon Green (OG) labeled c[KNGRE]-NH_2_ (OG attached to the side chain of Lys) revealed selective binding to CD13-receptor positive (CD13+) HT-1080 human fibrosarcoma cells and minimal binding to receptor negative (CD13−) MCF-7 human breast adenocarcinoma cells [15]. Moreover, a ^68^Ga- radiotracer labeled derivative of the cyclic [KNGRE]-NH_2_ has been successfully used for tumor diagnostic studies by PET, indicating its specific binding to CD13 receptor expressing tumor tissues [16].

Recently, we reported the synthesis and biochemical characterization of novel cyclic NGR peptides and their corresponding NGR-drug conjugates. Special attention was paid on the chemostability and in vitro biological activity of the compounds [17–18]*.* Daunomycin (Dau) was used as cytotoxic agent, attached to the NGR-derivatives via oxime linkage. The prepared conjugates revealed substantial in vitro cytostatic/cytotoxic effects. Our results indicated that the conjugates had an antitumor effect against both CD13+ HT-1080 cells and CD13− (but integrin receptor positive) HT-29 human colon cancer cells. Moreover, we showed that the toxicity and the selectivity of the conjugates highly depended on their structure, cellular uptake and propensity to deamidation.

**Figure 1:**
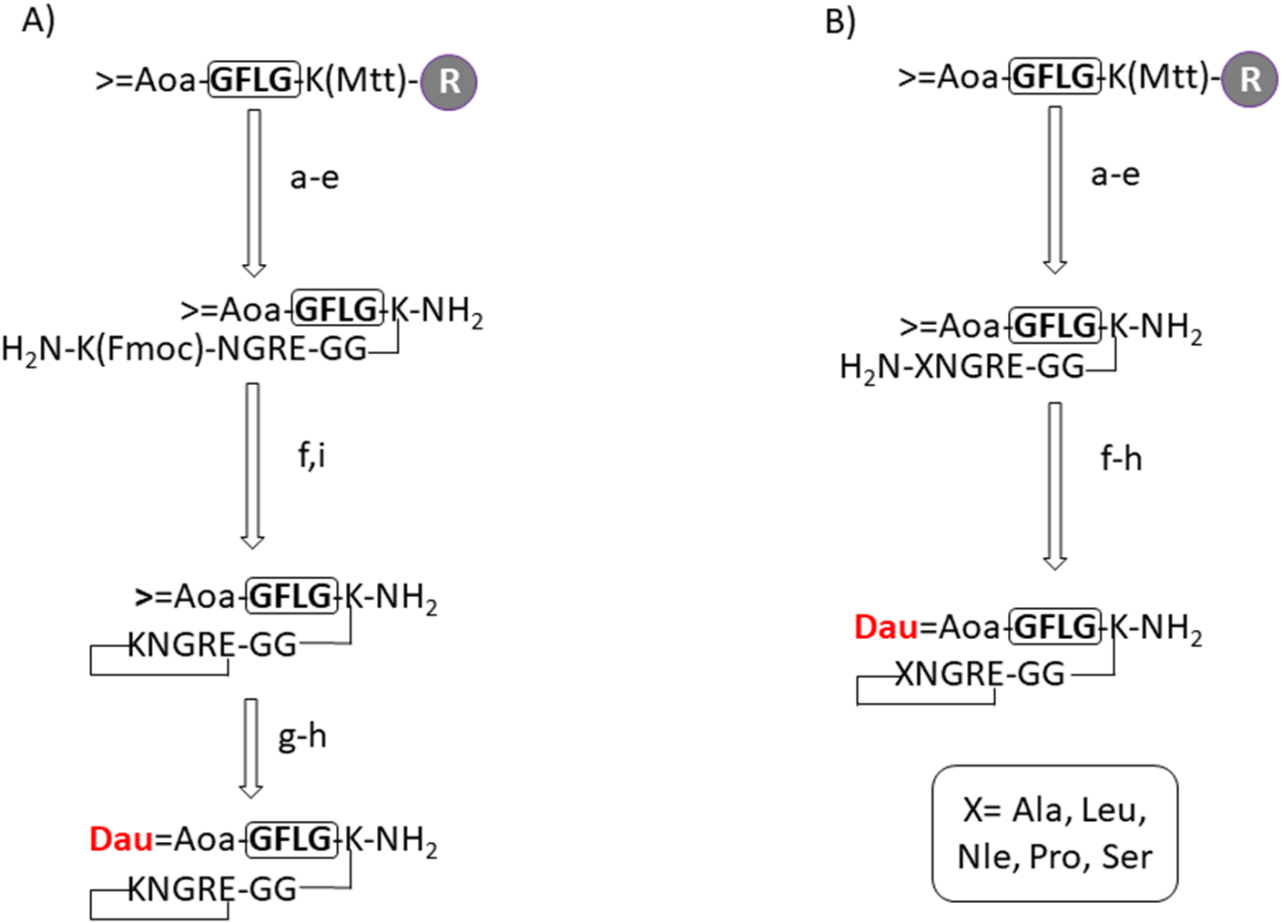
Schematic synthesis of cyclic KNGRE (A) and XNGRE (B) drug conjugates. a) Mtt-cleavage: 2% TFA/DCM; b) Fmoc-Aaa(X)-OH coupling; c) Fmoc-cleavage 2% piperidine/2% DBU/DMF, 0.1 M HOBt; d) cleavage from resin 2.5% TIS/2.5% H_2_O/95% TFA (rt, 3 h); e) salt exchange Pyr·HCl 10 equiv/MeOH (1 h); f) cyclization: BOP 3 equiv/HOBt 3 equiv/DIPEA 6 equiv/DMF (*c* = 0.5 mg/mL, rt, 24 h); g) deprotection of aminooxyacetic acid 0.2 M NH_4_OAc solution (pH 5.0)/1 M methoxylamine (rt, 1 h); h) daunomycin conjugation (rt, 24 h) in 0.2 M NH_4_OAc solution (pH 5.0); i) Fmoc-cleavage 4% hydrazine/DMF (rt, 2 h).

The most active conjugate with dual acting properties was Dau=Aoa-GFLGK(c[KNGRE]-GG-)-NH_2_ (**K**, control conjugate in this study). In this conjugate the cyclic NGR peptide was attached through a Gly-Gly dipeptide spacer to the lysine side chain connected to the chatepsin B labile GFLG spacer that allows lysosomal drug release. Dau was conjugated to the GFLG spacer via oxime linkage through an incorporated aminooxyacetyl (Aoa) moiety. The preparation of the conjugate required a sophisticated synthetic route and the use of orthogonal protecting groups (Figure 1A). Previous studies indicated that the free ε-amino group of Lys does not have an impact on the biological activity [15,17]. To prove our assumption, a set of novel cyclic NGR peptide–Dau conjugates were developed in which the Lys was replaced by different amino acids (Ala, Leu, Nle, Pro and Ser). The main goal of the present study was to investigate whether the exchange of the lysine in the cycle has any influence on the chemostability, selectivity and antitumor activity of the conjugates.

Here we report on the synthesis and characterization of the cyclic NGR peptide–Dau bioconjugates including chemostability, lysosomal degradation, cellular uptake studies and in vitro cytostatic/cytotoxic effect.

## Results and Discussion

### Synthesis of cyclic NGR–Dau conjugates

The NGR cyclic peptides were prepared as shown in Figure 1. All derivatives were synthesized by SPPS on a Rink-Amide MBHA Resin, using Fmoc/*t-*Bu strategy. The anticancer drug daunomycin was conjugated to the Aoa-GFLGK spacer via oxime linkage [17]. This spacer is degraded by lysosomal enzymes ensuring the release of the Dau=Aoa-Gly-OH as the smallest bioactive metabolite in lysosomes [19]. It is well known from our previous studies that not only the free Dau but also Dau containing metabolites like Dau=Aoa-Gly-OH bind to DNA efficiently resulting in antitumor activity. The exchange of the Lys in the cycle simplified the cyclization step and due to the avoidance of the Fmoc cleavage in solution (Figure 1B vs 1A) the compounds could be obtained in higher yields compared to the control (**K**). Isopropylidene protected aminooxyacetyl moiety was used to avoid unwanted reactions with aldehydes or ketones. This protecting group was removed with 1 M methoxylamine in 0.2 M NH_4_OAc solution (pH 5.0) prior to the Dau conjugation. The final cyclic NGR peptide–Dau conjugates were characterized by analytical HPLC and mass spectrometry (Table 1, Supporting Information File 1), whereby the purity was over 95% in all cases. In comparison with the control conjugate (**K**) significantly higher overall yield was observed in the case of conjugates **2**, **3** and **4** obtained with a lower yield. The improvement was observed especially in the cyclization step that might be explained by the lack of bulky protecting groups on amino acids used instead of Lys.

**Table 1:** List of the peptide drug-conjugates synthesized.

Code	Compounds	Yield^a^ (%)	RP HPLC *t*_R_ (min)^b^	ESIMS^c^ M(*calc*)/M(*exp*)

**1**	Dau=Aoa-GFLGK(c[CONH-ANGRE]-GG)-NH_2_	2.2	22.2	1725.8/1725.8
**2**	Dau=Aoa-GFLGK(c[CONH-LNGRE]-GG)-NH_2_	7.0	22.4	1767.8/1767.4
**3**	Dau=Aoa-GFLGK(c[CONH-NleNGRE]-GG)-NH_2_	10.6	22.6	1767.8/1767.2
**4**	Dau=Aoa-GFLGK(c[CONH-PNGRE]-GG)-NH_2_	6.2	17.3	1751.1/1751.4
**5**	Dau=Aoa-GFLGK(c[CONH-SNGRE]-GG)-NH_2_	1.8	20.1	1741.9/1741.7
**K**	Dau=Aoa-GFLGK(c[CONH-KNGRE]-GG)-NH_2_	2.0	23.0	1783.6/1783.0

^a^The overall yield was calculated for the starting amount and capacity of the resin. ^b^HPLC: KNAUER 2501; column: Phenomenex Luna C18 (250 mm × 4.6 mm, 5 µm silica, 100 Å pore size; gradient: 0 min 0% B; 5 min 0% B; 50 min 90% B; eluents: A) 0.1% TFA/water, B) 0.1% TFA/MeCN-H_2_O (80:20, v/v); flow rate: 1 mL/min; detection: λ = 220 nm. ^c^ESIMS: Bruker Daltonics Esquire 3000+ ion trap mass spectrometer; spectra were acquired in the 50–2500 *m/z* range.

### Chemostability of cyclic NGR peptide–daunomycin conjugates

The chemostability of cyclic NGR peptide–drug conjugates was studied under the treatment conditions used for the in vitro cytotoxicity experiments. Samples were taken at 0 min, 6 h and 72 h. The deamidation rate was evaluated by HPLC–MS. In contrast to the control conjugate (**K**) that showed high stability in our previous study, the new conjugates rearranged in time. The results showed very similar isoAsp/Asp (≈3:1) rates after deamidation of conjugates **1**, **2**, **3**, and **5** calculated from the area under the curve (Table 2, Supporting Information File 1, Figures S1–S5). After 6 h, moderate rearrangement was observed which increased in time. However, 54–58% of the parent cyclic NGR conjugates were still intact after 72 h. Lower stability was observed in the case of the Pro-containing conjugate (**4**) with faster deamidation and higher ratio of DGR. Except deamidation no other decomposition could be observed during this study.

**Table 2:** Chemostability of cyclic NGR peptide-Daunomycin conjugates.

Ratio of Asn-/Asp-/isoAsp-derivatives (DMEM CM, 37 °C)								
Code	AAA in position X of the conjugates	6 h				72 h		
				
		NGR	DGR	isoDGR		NGR	DGR	isoDGR

**1**	Ala	96	0	4		58	11	31
**2**	Leu	93	0	7		54	11	35
**3**	Nle	93	1	6		58	9	33
**4**	Pro	73	14	13		19	46	35
**5**	Ser	93	0	7		56	12	31
**K**	Lys	100	0	0		100	0	0

### Cytostatic/cytotoxic studies of NGR peptide–Dau conjugates

Similarly to our previous study the antitumor effects of conjugates were examined in vitro on CD13+ HT-1080 human fibrosarcoma and on CD13− HT-29 human colon adenocarcinoma cells. Both cell types are integrin receptor positive [17]. The effect of the new drug conjugates was compared with the toxicity of free Dau and our lead compound Dau=Aoa-GFLGK(c[KNGRE]-GG)-NH_2_ (**K**). The bioconjugates enter cancer cells most likely by receptor-mediated endocytosis (at least at lower micromolar concentration) followed by the release of the active metabolite Dau=Aoa-Gly-OH by lysosomal degradation. In contrast, free Dau enters the cells in an unspecific manner which might explain the lower antitumor effect of conjugates in comparison with the free drug. In this experiment we measured the cytostatic effect (6 h treatment and further 66 h incubation after washing out the compounds) and the cytotoxic effect (72 h treatment). The results are summarized in (Table 3). In contrast to **K** that is taken up by HT-1080 cells slightly more efficiently than by HT-29 and therefore shows higher antitumor effect against CD13+ cells, the new conjugates showed higher cytostatic/cytotoxic effects on the CD13− HT-29 colon cancer cells. It seems that the replacement of Lys by the hydrophilic amino acid Ser is not favorite. However, the incorporation of hydrophobic amino acids was well accepted. The conjugate with bulky side chain in this position (Leu) had higher IC_50_ values that might be explained by sterical hindrance. The conjugates with Ala or Nle showed the best antitumor activity on both cell lines. The Nle containing conjugate presented similar activity on HT-1080 and higher activity on HT-29 cells compared to the control conjugate. It is worth mentioning that Nle has a linear hydrocarbon side chain with the same length as Lys, the amino functional group missing. To further characterize the biological activity of the conjugates, their lysosomal degradation and cellular uptake were studied.

**Table 3:** In vitro cytostatic/cytotoxic effects of compounds on HT-29 and HT-1080 cells.

Compounds	HT-1080 (6 h) IC_50_ (μM)	HT-29 (6 h) IC_50_ (μM)	HT-1080 (72 h) IC_50_ (μM)	HT-29 (72 h) IC_50_ (μM)

Daunomycin	1.4 ± 0.6	0.3 ± 0.2	0.5 ± 0.2	0.1 ± 0.1
Dau=Aoa-GFLGK(c[KNGRE]-GG)-NH_2_ (**K**)	5.7 ± 0.5	8.7 ± 1.2	1.4 ± 0.7	3.0 ± 0.6
Dau=Aoa-GFLGK(c[ANGRE]-GG)-NH_2_ (**1**)	8.9 ± 0.8	4.3 ± 0.5	3.6 ± 0.7	3.2 ± 0.8
Dau=Aoa-GFLGK(c[LNGRE]-GG)-NH_2_ (**2**)	57.5 ± 6.3	47.0 ± 5.4	20.6 ± 0.4	14.1 ± 0.7
Dau=Aoa-GFLGK(c[NleNGRE]-GG)-NH_2_ (**3**)	5.5 ± 0.3	2.2 ± 0.2	2.3 ± 0.6	1.3 ± 0.2
Dau=Aoa-GFLGK(c[PNGRE]-GG)-NH_2_ (**4**)	9.4 ± 4.0	14.6 ± 4.7	3.5 ±1.0	3.7 ± 0.8
Dau=Aoa-GFLGK(c[SNGRE]-GG)-NH_2_ (**5**)	>100	64.7 ± 4.9	63.7 ± 9.5	39.4 ± 2.9

In this study our goal was to compare the in vitro antitumor activity of the conjugates. We believe that the measurement of binding affinity on isolated receptors, that was not task of this experiment, could not explain properly the efficacies and selectivity. The receptor profile of both cell types are very complex. HT-1080 contain different integrins (RGD, collagen, etc) next to CD13 receptor [20]. HT-29 expresses all the known β_1_ RGD dependent receptors furthermore α_v_β_3_, α_v_β_5_, α_v_β_6_, α_v_β_8_ [21]. Most of the mentioned integrins bind isoDGR peptides with different affinity from nM up to μM concentration that depends on the structure of the peptide [2]. In addition the binding affinity of the different integrins is not consequent to the peptides. Furthermore, there are only a few binding affinity studies for CD13 suggesting several hundred nM IC_50_ values for NGR peptide derivatives [22]. The biological activity of NGR and isoDGR peptides might be influenced also by the density of the different receptors on the tumor cells, which is not so easy to identify in case of such complex receptor profile. Therefore, the resulted amount of isoDGR derivatives might provide similar activity both on CD13+ and CD13− cells.

### Lysosomal degradation

Lysosomal degradation studies were carried out as previously described [17]. The results showed that all conjugates decomposed within 6 h (Supporting Information File 1, Figures S6–S10). The main cleavage site of the conjugates could be detected between Gly-Phe within the enzyme labile spacer resulting in the smallest active Dau containing metabolite Dau=Aoa-Gly-OH. No significant difference in degradation speed of the conjugates was observed. Therefore, the replacement of Lys has no influence on the biological activity through the lysosomal degradation.

### Cellular uptake

**Figure 2:**
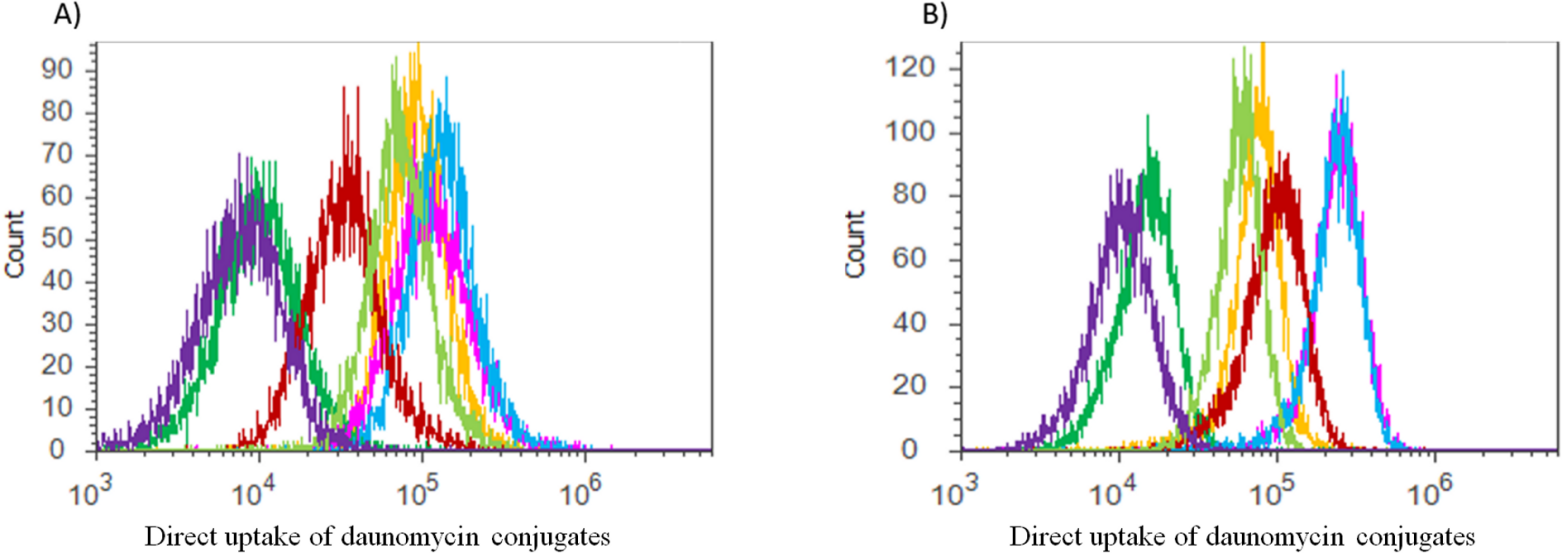
(A) HT1080 and (B) HT-29 cells. Uptake of conjugate **1** (light green); **2** (red); **3** (light blue); **4** (pink); **5** (green); **K** (yellow). Empty control with purple color.

Daunomycin is fluorescent therefore the cellular uptake of Dau containing conjugates can be followed by flow cytometry (Figure 2). The new conjugates were taken up by HT-29 cells in a higher amount than by HT-1080 cells. Conjugates **2**, **3** and **4** showed significantly higher accumulation in HT-29 than in HT-1080 cells compared to the other conjugates. The highest uptake by both cell types was observed in the case of conjugates **3** and **4**. The low cytostatic/cytotoxic effects of conjugate **5** can be explained by the results of cellular uptake study. The Ser-containing conjugate did not enter HT-1080 cells, while a slightly higher cellular uptake was detected in HT-29 cells, although this uptake was still much lower than in the case of the other conjugates.

## Conclusion

From this study we can conclude that replacement of Lys in the Dau=Aoa-GFLGK(c[KNGRE]-GG)-NH_2_ conjugate by different amino acids provides a more convenient and cost-effective synthetic route resulting in a higher yield, which might be relevant for larger scale synthesis needed for further in vivo studies. We show that the changes decrease the chemostability of the cyclic NGR moiety, resulting in the formation of isoAsp derivatives in higher amount. Among the new cyclic NGR peptide–daunomycin conjugates the most effective compound was Dau=Aoa-GFLGK(c[NleNGRE]-GG)-NH_2_, which showed similar activity against HT-1080 CD13+ cells to Dau=Aoa-GFLGK(c[KNGRE]-GG)-NH_2_, and a significantly higher antitumor effect against HT-29 CD13− but integrin receptor positive cells. This might be explained by the binding affinity of isoDGR peptides to integrin receptors. However, to confirm this findings further binding studies of cyclic NGR peptide–drug conjugates to different integrin receptors are needed. Taken together, the synthetic and biological results suggest that the Dau=Aoa-GFLGK(c[NleNGRE]-GG)-NH_2_ conjugate is more suitable for drug targeting with dual acting propensity than our control lead compound.

## Experimental

### Synthesis of the novel peptide–drug conjugates

Linear precursor peptides were prepared on Rink-Amide MBHA resin by SPPS. Standard Fmoc protected amino acids (Iris Biotech GmbH, Marktredwitz, Germany) were used for the synthesis except Fmoc-Lys(Mtt)-OH that was applied for the development of branching in the peptide. The protocol of the SPPS was similarly as described in [17] as follows: (i) DMF washing (4 × 0.5 min), (ii) Fmoc deprotection with 2% DBU, 2% piperidine, 0.1 M HOBt in DMF (4 times; 2 + 2 + 5 + 10 min), (iii) DMF washing (10 × 0.5 min), (iv) coupling of Fmoc-protected amino acid derivative: DIC:HOBt in DMF (4 equiv each) (1 × 60 min), (v) DMF washing (3 × 0.5 min), (vi) DCM washing (2 × 0.5 min), (vii) ninhydrin or isatin test. The cleavage of Mtt protecting group was achieved by using 2% TFA in DCM for 6 × 4 min. The coupling of the isopropylidene protected aminooxyacetic acid [17] to the *N*-terminus of the linker sequence was carried out by using standard protocol DIC/HOBt coupling. The cleavage from the solid support was performed at rt in a solution of 95% TFA, 2.5% triisopropylsilane, and 2.5% water for 3 h. The resin was then filtered and the crude product was precipitated with cold diethyl ether and pellet centrifugated for 5 min at 4000 rpm. After washing (3 times with ether) the remaining pellet was dissolved in water and lyophilized. The lyophilized compound was then purified by RP-HPLC prior to the cyclization.

### Cyclization

Prior to the head-to-side chain cyclization a salt exchange needs to be performed using 10 equiv of pyridinium hydrochloride in 5 mL MeOH. After 20 min volatiles were removed under reduced pressure, the remaining oily compound was dissolved in dry DMF at a concentration of 0.5 mg/mL. The pH of the solution was adjusted to pH 8 with DIPEA, then BOP and HOBt (3 equiv each) are added to the mixture. The reaction was followed by analytical HPLC till the complete conversion. At the end DMF was removed and the oily compound was dissolved in acetonitrile–water and purified by RP-HPLC and the collected fractions lyophilized.

### Purification

Analogous to the description in [17] RP-HPLC purification was used for the isolation of pure peptides and conjugates. A KNAUER 2501 HPLC system (KNAUER, Bad Homburg, Germany) was applied with a semi-preparative Phenomenex Luna C18 column (250 mm × 21.2 mm) with 10 µm silica (100 Å pore size) (Torrance, CA). Linear gradient elution (0 min 15% B; 5 min 15% B; 55 min 70% B) with eluent A (0.1% TFA in water) and eluent B (0.1% TFA in MeCN–H_2_O (80:20, v/v)) was used at a flow rate 9.5 mL/min. Peaks were detected at 220 nm.

### Chemostability studies

As described in [17] to check the chemical stability each of the drug conjugates were dissolved in DMSO (2% of the final volume), following the addition of 10% FBS (fetal bovine serum) containing complete cell culture medium (DMEM CM) up to the 1 mg/mL final concentration. Each conjugate was allowed to incubate at 37 °C. Samples were analyzed at experiment time of 0 h, 6 h and 72 h, respectively, and components were purified with Amicon Ultra Centrifugal Filters (cut off 10K Millipore). The membranes were washed with eluent A (HPLC), followed by 3 × 15 min centrifugation at 13000 rpm. The last step is the washing with eluent B (HPLC) 1 × 15 min, followed by lyophilization and concentration of the samples.

### In vitro cytostatic effect and cytotoxicity

HT-1080 was maintained in DMEM while HT-29 cells in RPMI (Sigma-Aldrich), respectively, supplemented with 10% FBS, 5 mmol/L glutamine, and 50 units/mL penicillin and streptomycin (Life Technologies). As described in [17] cells were seeded in 5000 cells/well density in 100 μL medium followed by an overnight incubation. The next day 100 μL of serially diluted drugs were added to the cells. For the measurements of cytostatic effect, drug containing medium was gently removed from the plates after 6 h incubation, fresh medium was added to each wells, and the plates were further incubated for additional 66 h (72 h in total). In the case of cytotoxicity measurements, the drug containing medium was on the cells for the full period of the 72 h assay. At 72 h, supernatant was removed from the cells, and viability was assessed by the PrestoBlue^®^ reagent (Life Technologies), which was diluted in PBS to reach the concentration given in the manufacturer’s instruction.

### Lysosomal degradation

Rat liver lysosomal homogenate was prepared for this experiment. The protein concentration was detected with bicinchoninic acid (Pierce BCA protein assay) following the manufacturer’s protocol (ThermoFischer Scientific, Rockford, IL, USA), and it was 17.4 μg/µL. The peptide–drug conjugates were dissolved in deionized water (4 μg/mL concentration). The solutions were further diluted to a concentration of 0.2 μg/µL using 0.2 M of NH_4_OAc (pH 5.0). The lysosomal homogenate was diluted with 0.2 M NH_4_OAc (pH 5.0) to a concentration of 3.48 µg/µL. The homogenate was then added to the conjugates in a ratio of 1:1 w/w. All the degradation mixtures were kept at 37 °C, samples of 13 µL were taken at 0 h, 6 h and 72 h. Reaction mixtures were quenched by the addition of 2 µL of formic acid. LC–MS analysis was performed at the end on each sample.

### Cell uptake

Analogous to the description in [17], prior to the treatment, HT-1080 and HT-29 cells were incubated overnight in cell culture medium (see above) followed by seeding at a 250.000 cells/well density. Peptide–drug conjugates were dissolved in FBS containing cell culture medium and added to the cells at 10 μM final concentration. After 6 h incubation at 37 °C the supernatant was removed, cells were washed with PBS and trypsinised with 0.1% trypsin (Gibco^®^ by Life Technologies) for 10 minutes. Trypsinization was terminated with FBS containing medium, then cells were washed and suspended in serum free medium. For live/dead detection we used Zombie Violet reagent (Biolegend, San Diego CA). Samples were detected and analyzed by using an Attitude^®^ Acoustic Focusing Cytometer (ThermoFischer Scientific).

## Supporting Information

File 1 Chemo stability and lysosomal degradation measurements.
