# Supplementary material for: Development of novel cyclic NGR peptide–daunomycin conjugates with dual targeting property
Source: Beilstein J Org Chem. 2018 Apr 25;14:911–8. doi: 10.3762/bjoc.14.78 (PMC5942373; doi:10.3762/bjoc.14.78)

Supporting Information

for

Development of novel cyclic NGR peptide–daunomycin conjugates with dual targeting property

Andrea Angelo Pierluigi Tripodi[1,2], Szilárd Tóth[3], Kata Nóra Enyedi[1,2], Gitta Schlosser[1,2], Gergely Szakács[3,4] and Gábor Mező[1,2*]

Address: [1]Eötvös Loránd University, Faculty of Science, Institute of Chemistry, Pázmány P. stny. 1/A, H-1117 Budapest, Hungary, [2]MTA-ELTE Research Group of Peptide Chemistry, Hungarian Academy of Sciences, Eötvös Loránd University, Pázmány P. stny. 1/A, H-1117 Budapest, Hungary, [3]Institute of Enzimology, Research Center for Natural Sciences, Hungarian Academy of Sciences, Magyar tudósok körútja 2, H-1117 Budapest, Hungary and [4]Institute of Cancer Research, Medical University Vienna, Borschkegasse 8a, A-1090 Vienna, Austria

Email: Gábor Mező - gmezo@elte.hu

*Corresponding author

Chemo stability and lysosomal degradation measurements

Table of contents

Figure S1) Chemo stability of conjugate 1: Dau=Aoa-GFLGK(c[CONH-ANGRE]-GG)-NH_2......................S2

Figure S2) Chemo stability of conjugate 2: Dau=Aoa-GFLGK(c[CONH-LNGRE]-GG)-NH_2......................S2

Figure S3) Chemo stability of conjugate 3: Dau=Aoa-GFLGK(c[CONH-NleNGRE]-GG)-NH_2..................S3

Figure S4) Chemo stability of conjugate 3: Dau=Aoa-GFLGK(c[CONH-PNGRE]-GG)-NH_2......................S3

Figure S5) Chemo stability of conjugate 3: Dau=Aoa-GFLGK(c[CONH-SNGRE]-GG)-NH_2......................S4

Figure S6) Lysosomal degradation of Conjugate 1: Dau=Aoa-GFLGK(c[CONH-ANGRE]-GG)-NH_2.........S4

Figure S7) Lysosomal degradation of Conjugate 2: Dau=Aoa-GFLGK(c[CONH-LNGRE]-GG)-NH_2.........S5

Figure S8) Lysosomal degradation of Conjugate 3: Dau=Aoa-GFLGK(c[CONH-NleNGRE]-GG)-NH_2......S5

Figure S9) Lysosomal degradation of Conjugate 2: Dau=Aoa-GFLGK(c[CONH-PNGRE]-GG)-NH_2..........S6

Figure S10) Lysosomal degradation of Conjugate 2: Dau=Aoa-GFLGK(c[CONH-SNGRE]-GG)-NH_2........S6

Chemo stability measurements:

Figure S1) Chemo stability of conjugate 1: Dau=Aoa-GFLGK(c[CONH-ANGRE]-GG)-NH_2

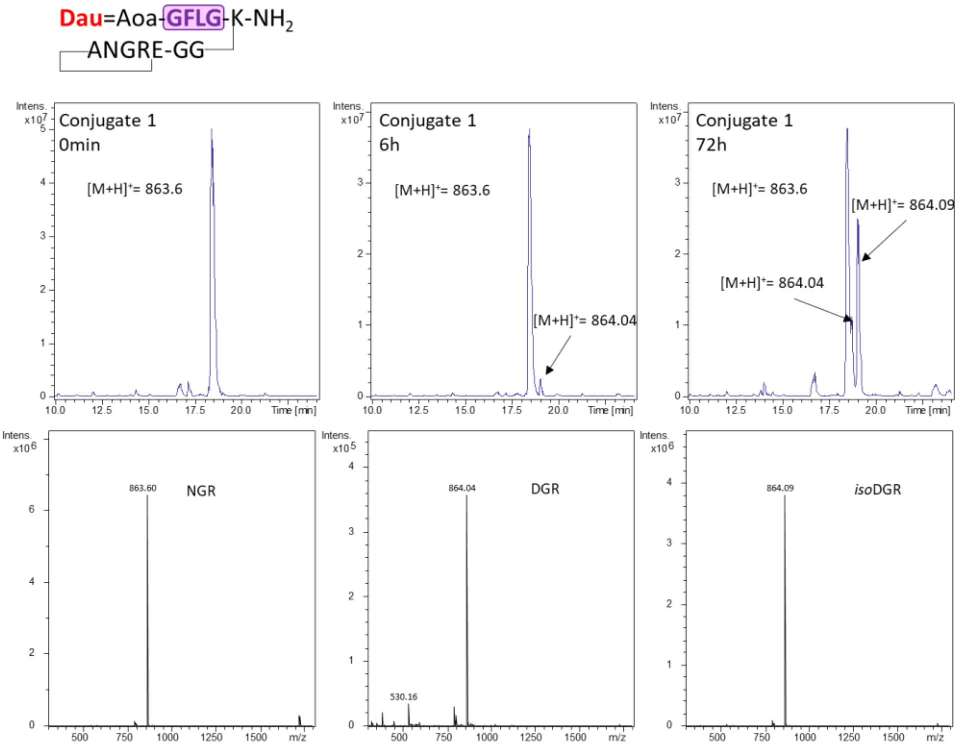

Figure S2) Chemo stability of conjugate 2: Dau=Aoa-GFLGK(c[CONH-LNGRE]-GG)-NH_2

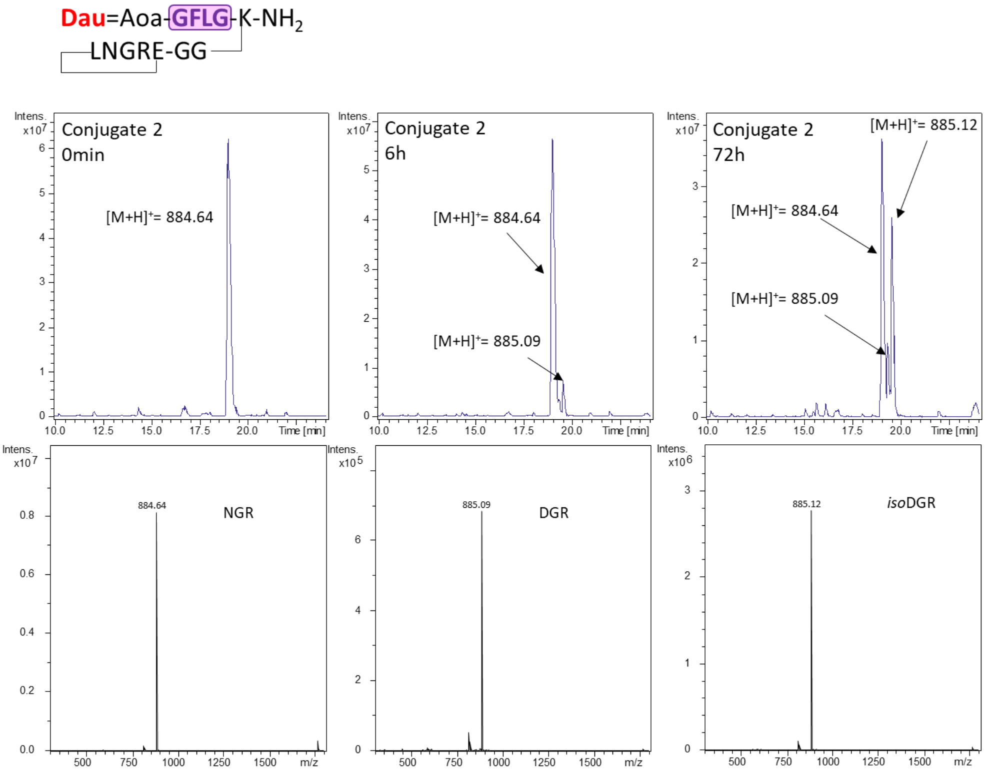

Figure S3) Chemo stability of conjugate 3: Dau=Aoa-GFLGK(c[CONH-NleNGRE]-GG)-NH_2

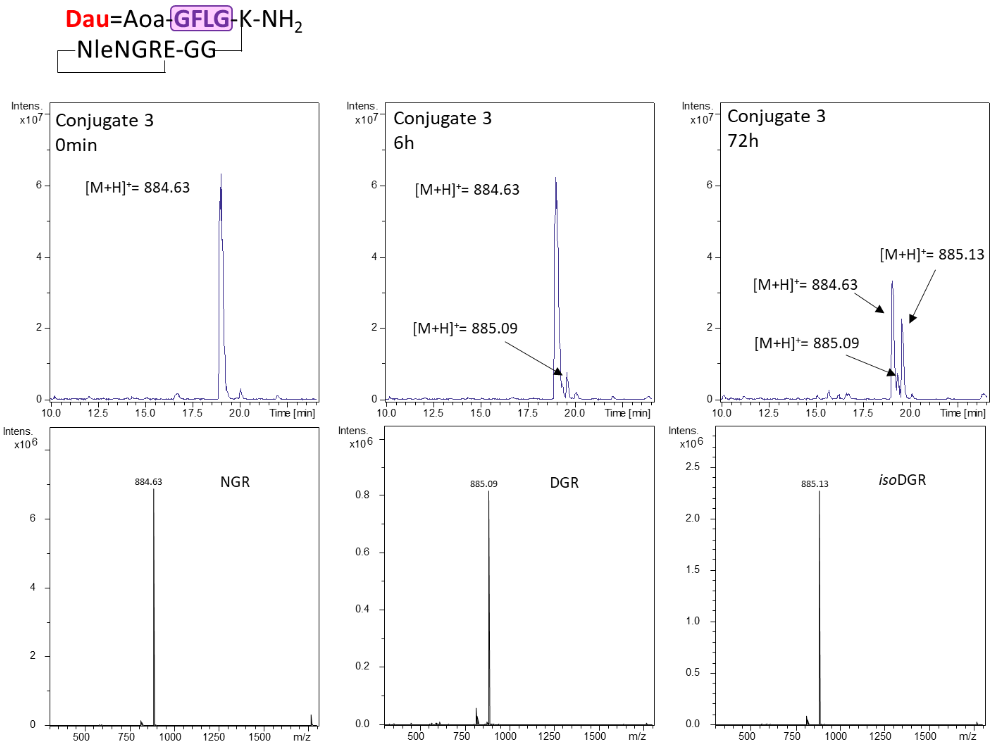

Figure S4) Chemo stability of conjugate 3: Dau=Aoa-GFLGK(c[CONH-PNGRE]-GG)-NH_2

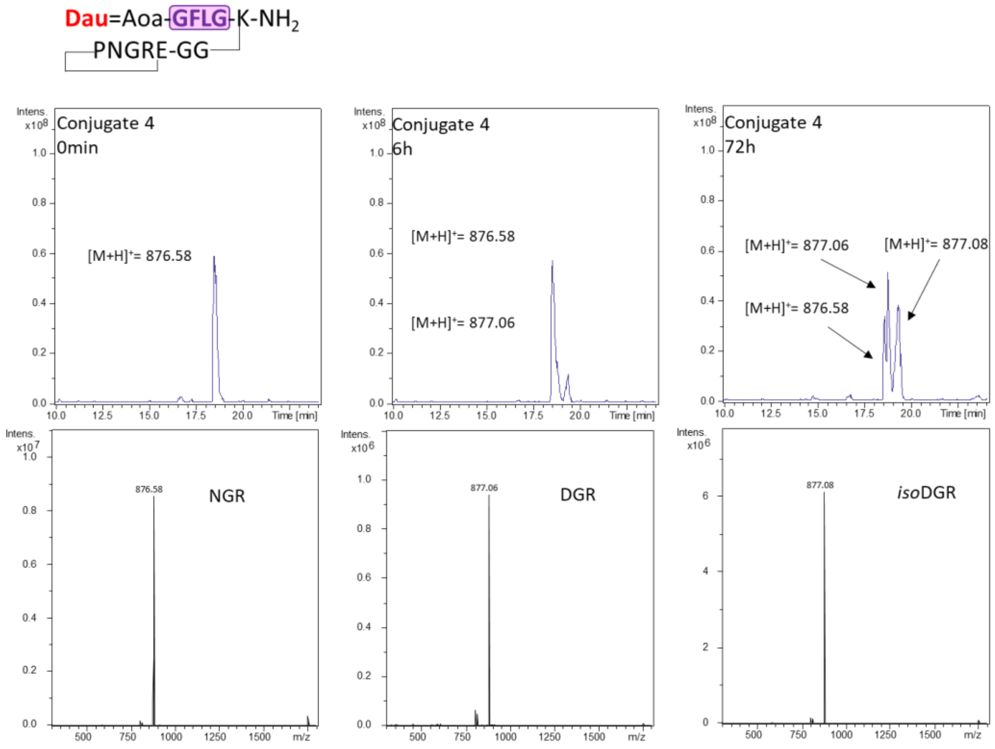

Figure S5) Chemo stability of conjugate 3: Dau=Aoa-GFLGK(c[CONH-SNGRE]-GG)-NH$_2$

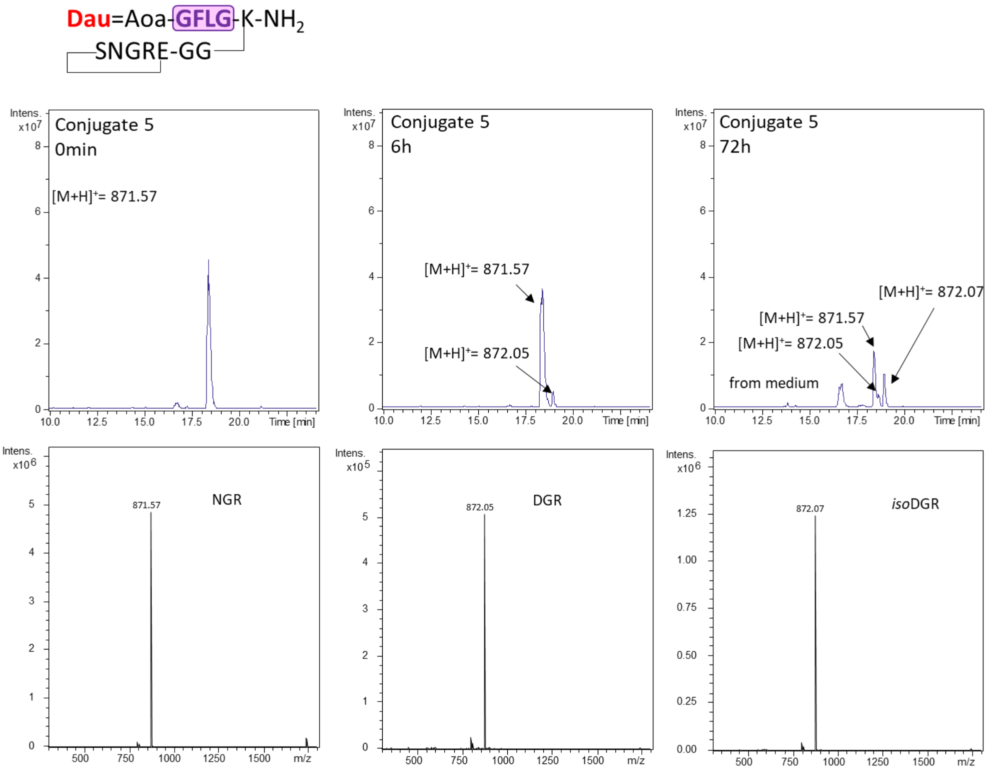

Figure S6) Lysosomal degradation of conjugate 1: Dau=Aoa-GFLGK(c[CONH-ANGRE]-GG)-NH$_2$

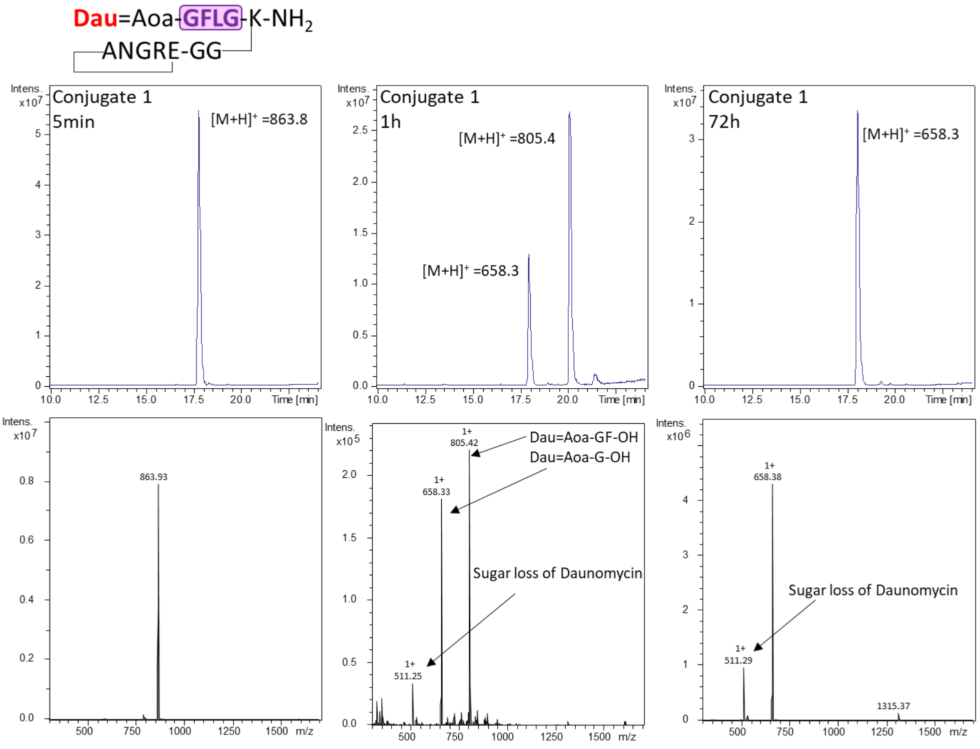

Figure S7) Lysosomal degradation of conjugate 2: Dau=Aoa-GFLGK(c[CONH-LNGRE]-GG)-NH$_2$

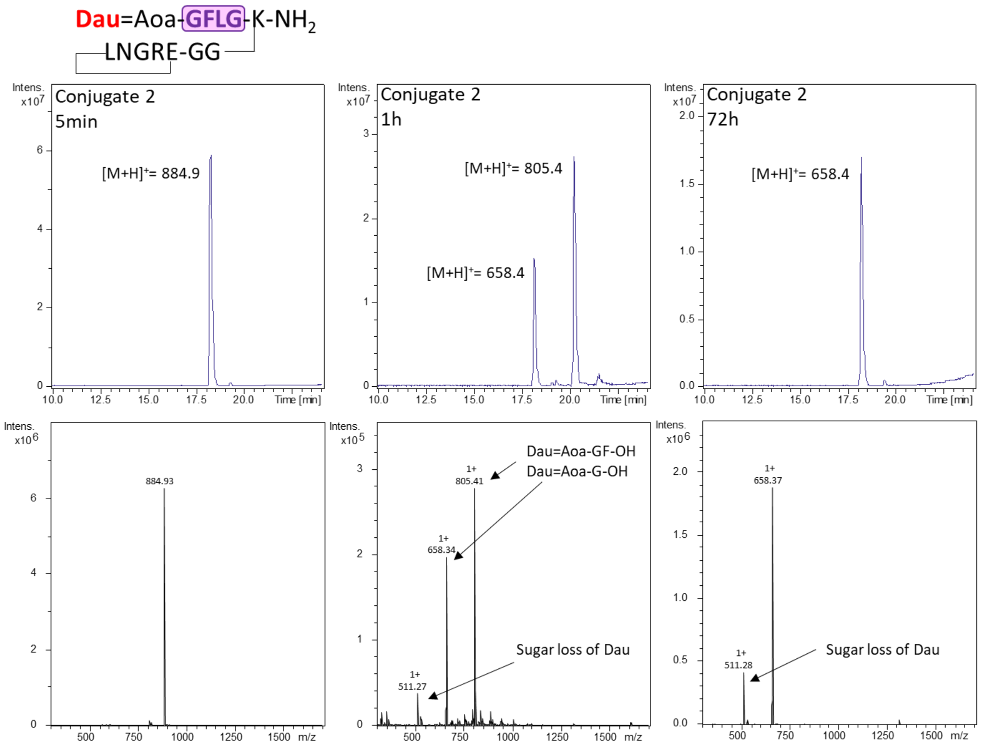

Figure S8) Lysosomal degradation of conjugate 3: Dau=Aoa-GFLGK(c[CONH-NleNGRE]-GG)-NH$_2$

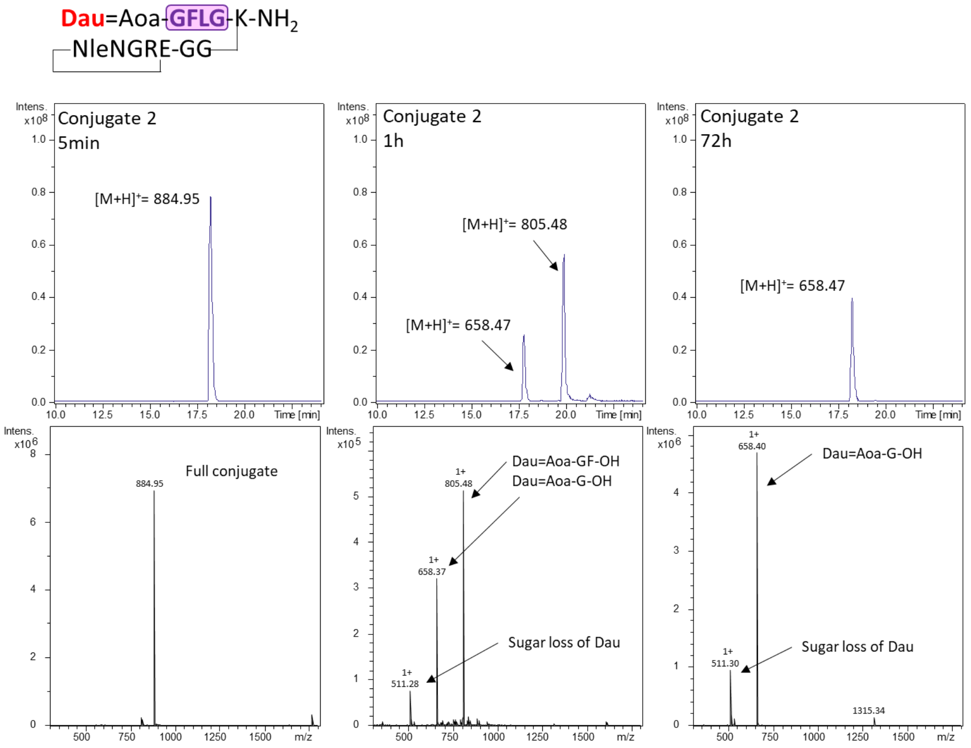

Figure S9) Lysosomal degradation of conjugate 2: Dau=Aoa-GFLGK(c[CONH-PNGRE]-GG)-NH$_2$

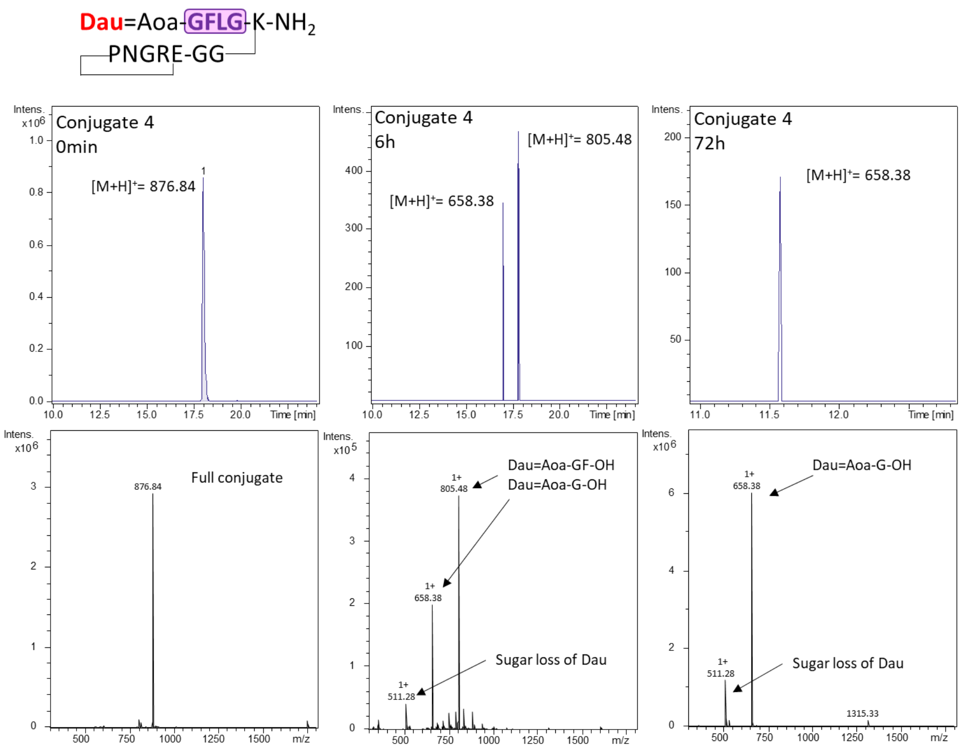

Figure S10) Lysosomal degradation of conjugate 2: Dau=Aoa-GFLGK(c[CONH-SNGRE]-GG)-NH$_2$

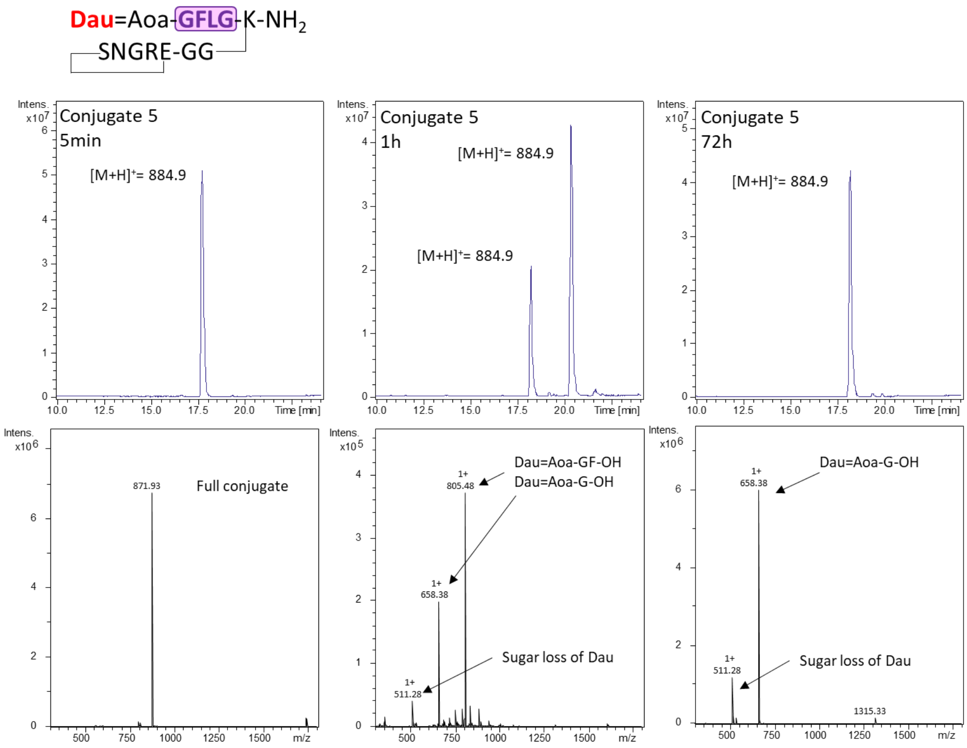

Supplement: File 1 — Chemo stability and lysosomal degradation measurements. [file Beilstein_J_Org_Chem-14-911-s001.pdf]
